# Integrating Gender-Based Violence Services Into HIV Care: Insights From Malawi

**DOI:** 10.9745/GHSP-D-24-00177

**Published:** 2025-08-14

**Authors:** Christine Kiruthu-Kamamia, Evelyn Viola, Odala Sande, Tapiwa Kumwenda, Joseph Lungu, Joseph Diele, Ellen MacLachlan, Agnes Thawani

**Affiliations:** a Lighthouse Trust, Lilongwe, Malawi.; b International Training and Education Center for Health, University of Washington Department of Global Health, Seattle WA, USA.

## Abstract

**Introduction:**

Gender-based violence (GBV) not only poses significant public health and human rights challenges but is also closely associated with HIV. GBV acts as a barrier to HIV prevention, testing, and treatment adherence, and fear of GBV inhibits disclosure of HIV status to sexual partners. In Malawi, where both GBV and HIV prevalence is high, integrating GBV services into HIV care is crucial. We describe the integration of GBV services into Lighthouse Trust’s HIV testing and treatment clinics in Malawi, including screening, documentation, intervention implementation, outcomes, and lessons learned.

**Methods:**

We conducted a retrospective analysis from January 2020 to June 2024. Data on cases identified, post-GBV services, and perpetrator demographics were collected from the GBV register. We used descriptive statistics to describe the intervention outcomes.

**Results:**

We documented 9,045 reported GBV cases among males and females from January 2020 to June 2024. Adolescent girls aged 10–19 years constituted a significant proportion of survivors. Psychosocial services were the most common type of service that was offered to GBV survivors (25%), followed by HIV testing (19%) and sexually transmitted infection screening (18%). Perpetrators were mostly known to survivors.

**Conclusion:**

We successfully integrated GBV services into the Lighthouse Trust HIV clinics in close collaboration with the one-stop centers in Malawi. Training health care providers enhanced support for GBV survivors, with a focus on increasing awareness, especially for children and adolescents. Recommended actions include improving access to GBV services, enhancing documentation, and promoting multi-sectoral collaboration to ensure comprehensive care aimed at creating a safer, more dignified health care environment for all, particularly GBV survivors.

## Abstract (precis)

We describe the integration of gender-based violence (GBV) services into HIV services to address the intersecting needs of individuals affected by GBV and HIV.

## Teaser

**Key Findings:**

- Integrating gender-based violence (GBV) services into HIV care entailed engaging stakeholders to ensure the integration process addressed the needs of GBV survivors, providing adequate health care provider training to identify GBV and provide support and care, developing standardized procedures for screening and identification of GBV cases, strengthening documentation of cases, and enhancing referral procedures for post-GBV care services.
- From January 2020 to June 2024, over 9,000 cases of GBV were reported, and over 18,00 post-GBV services were documented.
- Of the GBV cases reported, 54% were among adolescents aged 10–19 years, 92% were female survivors, and 45% were incidents of sexual violence.

**Key Implications:**

- We provide a replicable model for health care providers and facility administrators, demonstrating the effectiveness of standardized screening, care, and referral processes in improving the support and outcomes for GBV survivors within HIV care in low-resource settings.
- Our findings can inform the development of more inclusive health policies and support services in HIV care that address the needs of all GBV survivors.

## BACKGROUND

Gender-based violence (GBV) is defined as violence that results or may result in physical, sexual, or psychological harm or suffering to individuals based on their gender.^1^ GBV is not only a major public health problem but also a human rights issue, deeply rooted in structural, gender-based inequalities perpetuated by social and political forces.^2^ The 4 main types of GBV—physical, sexual, emotional or psychological, and economic—are linked to a broad range of mental and physical health problems, including HIV.^3^

In Malawi, GBV remains pervasive, with all 4 types presenting in varying degrees.^4^ A 2013 survey found that 1 in 5 females and 1 in 7 males in Malawi have experienced at least 1 incident of sexual abuse before the age of 18 years.^5^ Given Malawi’s high HIV prevalence at 8.9% among adults,^6^ addressing the intersection of GBV and HIV/AIDS is particularly critical. Research shows that exposure to GBV is closely associated with poor health outcomes,^7^ including HIV acquisition and treatment interruption.^8^ GBV acts as a barrier to HIV prevention, testing, and treatment adherence, with fear of GBV inhibiting disclosure of HIV status to sexual partners.^9^^–^^11^ In addition, HIV is associated with increased experience with GBV, where women living with HIV have reported violence after disclosing their status,^12^ including reproductive coercion (forced or coerced sex, sabotage of contraception, or the forcible control of reproductive health by an abusive partner).^13^^–^^15^

To support GBV survivors, the government of Malawi established one-stop centers (OSCs) in select hospitals, providing comprehensive post-GBV care, including clinical, psychosocial, and legal support.^16^ These OSCs house health care providers, social workers, and police in the same location to manage GBV cases. Despite the establishment of OSCs with designated space and staff to support GBV survivors, challenges persisted, including the lack of a comprehensive data collection tool, incomplete documentation, and staffing shortages that hindered consistent operations. In addition, some guardians hesitated to report sexual violence cases among minors if they perceived the encounter as consensual or if the perpetrator was someone they relied on financially. This highlights a critical gap in awareness and education around GBV.

Many GBV interventions focus on identifying and reducing partner violence in antenatal care or primary health care settings.^17^^,^^18^ However, few studies have evaluated comprehensive GBV integration within HIV services across the care continuum.^19^^–^^22^ Routine screening for GBV in health care settings is not recommended unless under certain circumstances.^7^^,^^23^ However, some studies have shown that women are receptive to GBV screening, and it can help identify those at risk of violence.^24^ Given that HIV increases the risk of GBV,^25^ it is essential to integrate GBV services into HIV care to address the intersecting needs of individuals affected by both HIV and GBV.

Integrating GBV services into HIV care requires a coordinated approach that addresses both the health and psychological needs of survivors.^7^ Best practices for such integration in low-resource settings are limited, but lessons learned from integrating GBV response into HIV services can guide health care providers in effectively incorporating GBV screenings, referrals, and care into HIV services. These insights can shape protocols, inform training programs, and influence policy, ensuring comprehensive care for all GBV survivors.

Integrating GBV services into HIV care requires a coordinated approach that addresses both the health and psychological needs of survivors.

In this article, we describe the integration of GBV services at the Lighthouse Trust HIV clinics in Malawi by analyzing routinely collected data. We detail the GBV screening process, documentation, intervention implementation, best practices, and lessons learned, as well as the uptake of GBV services. By examining this integration, we aim to contribute to the existing body of knowledge and inform future interventions and policies to address GBV effectively within HIV care.

## METHODS

### Setting

The Lighthouse Trust is a World Health Organization (WHO)-recognized Center of Excellence (COE) in HIV management and the largest HIV clinic in Malawi.^26^^–^^28^ The Lighthouse Trust operates 5 integrated HIV testing, treatment, and care COEs across Malawi. One COE is in the Martin Preuss Center at Bwaila Hospital in Lilongwe, and 4 are strategically located in tertiary central hospitals where OSCs have been established: 1 at the Lighthouse Center at Kamuzu Central Hospital (KCH) in Lilongwe, 1 at the Umodzi Family Center at Queen Elizabeth Central Hospital in Blantyre, 1 at the Tisungane Clinic at Zomba Central Hospital in Zomba, and 1 in Mzuzu at Rainbow Clinic at Mzuzu Central Hospital.^29^ Additionally, the Lighthouse Trust supports 8 Ministry of Health-operated health centers in Lilongwe, including Area 18, Chileka, Chitedze, Kawale, KCH, Lumbadzi, Mitundu, and Nathenje.^29^

### Integration of GBV Services Into Lighthouse Centers of Excellence

Lighthouse Trust began offering GBV services in January 2020. To ensure efficient delivery of GBV services, from October to December 2019, Lighthouse Trust conducted several activities as part of the integration, including engaging stakeholders, training health care providers, developing a standard operating procedure, enhancing referral for post-GBV care, and developing data collection tools.

#### Stakeholder Engagement

Key stakeholders, including health care providers, community leaders, police, and GBV survivors, were engaged to ensure the integration process was inclusive and addressed the multifaceted needs of survivors.

#### Trained Health Care Providers

Providers underwent training to recognize subtle behaviors, health issues, physical and emotional signs, or services sought that might indicate the presence of GBV (Supplement 1). Three distinct training programs were implemented to enhance the capabilities of key health care providers, including nurses, clinicians, HIV diagnostic assistants, and psychosocial counselors. The first training was standardized training focused on GBV and the OSC function and conducted by facilitators from the Ministries of Gender, Health, and the Judiciary, and the police. The second training program was not standardized but addressed GBV and child protection and was conducted by the Police-Victim Support Unit. The third training was on using the LIVES (Listen, Inquire, Validate, Enhance safety, and Support)^17^ model, an initiative by WHO aimed to assist providers in identifying instances of violence within a clinical setting and providing initial support. This training was conducted by 2 Lighthouse Trust GBV team leads who were trained as trainers through a standardized U.S. Centers for Disease Control and Population training on LIVES. These comprehensive training sessions sought to equip health care providers with a holistic understanding of survivor management. The trainings included various topics, such as describing different forms of GBV, GBV screening using a tool adapted from Papua New Guinea,^30^ offering initial support, guidelines for reporting incidents to the police, child rights, comprehensive health care packages, forensic examinations, courtroom testimony, and proper documentation in the GBV register. In total, 174 providers were trained across all COEs. Refresher training using the LIVES model was then conducted annually. Feedback from providers indicated that refresher training was essential to reinforce skills and update knowledge on new protocols.

#### Standard Operating Procedure and Intensified Screening

Based on WHO guidelines,^8^ Lighthouse Trust did not conduct universal screening for GBV. Screening was only conducted when providers identified GBV indications to a recipient of care. A GBV survivor could be identified at any service delivery point (e.g., clinic room, reception area, vitals area, or HIV testing room) through several means: self-description of assault/violence, a referral letter from police, or clinical suspicion (e.g., through complaints or excuses, identified only in clinic rooms). To ensure the accuracy and consistency of GBV screening procedures, Lighthouse Trust developed a detailed standard operating procedure used exclusively in Lighthouse Trust facilities (Supplement 2) that outlined the appropriate protocols for administering GBV screening. GBV screening was also introduced in all clinic service delivery points, including the front desk and guard stations, where health care workers used a standardized screening tool (Supplement 3) when they suspected potential signs and symptoms of GBV among clients.

#### Enhanced Referral for Post-GBV Care

All clients who were identified as having experienced GBV were referred to the appropriate provider for post-GBV care. Before making the referral, the client was given adequate time to decide whether to accept the referral. Providers did not exert pressure on clients who refused referral services but instead provided information on available post-GBV care and educated the client on the mental and physical health effects of GBV. Based on the type of GBV, Lighthouse Trust provided the following clinical services: HIV testing, post-exposure prophylaxis (PEP), syndromic sexually transmitted infections (STI) screening and treatment, psychosocial services (PSS) (Supplement 4), and provision of emergency contraception. In cases where the client had experienced severe physical violence, the client was referred to the central hospital for advanced medical care. Furthermore, the client was referred to the OSC, where a police officer provided judicial support. A follow-up visit was then scheduled for all clients if they were willing.

#### Data Collection Tools

**FIGURE 1:**
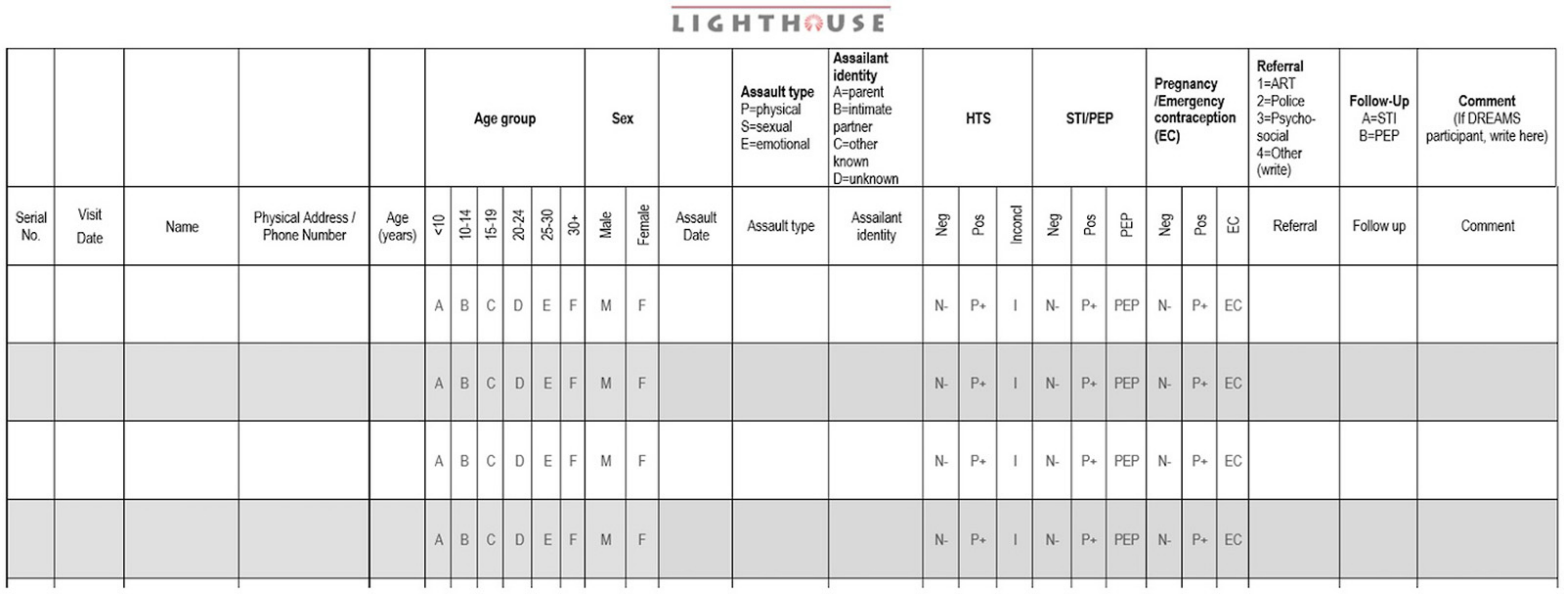
Register Used to Record Gender-Based Violence Cases in Services Integrated With HIV Services, Malawi

The OSCs did not possess an official register to record GBV cases before the service integration. Instead, cases were documented in a hardcover notebook that was used for several years, resulting in a nearly torn cover. Furthermore, available data and information gaps, such as missing client data on types of GBV experienced and post-GBV services provided, indicated the need to enhance GBV documentation processes. To address this issue, Lighthouse Trust developed a comprehensive and user-friendly GBV register incorporating essential indicators for both the OSC and Lighthouse Trust (Figure 1).

### Data Collection and Analysis

Data were collected from the Lighthouse Trust GBV registers from January 2020 to June 2024. All data were deidentified before extraction for data analysis. All Lighthouse Trust staff signed a mandatory confidentiality agreement as a condition of employment. A retrospective analysis was conducted using descriptive statistics to analyze the data using Microsoft Excel.

### Ethical Approval

The review was conducted using routinely collected data. Lighthouse Trust obtained Institutional Review Board approval from the National Health Science Research Committee (NHSRC) under protocol 2812, which covers the use of these data.

## RESULTS

### Gender-Based Violence Cases and Trends in Clinics

**FIGURE 2:**
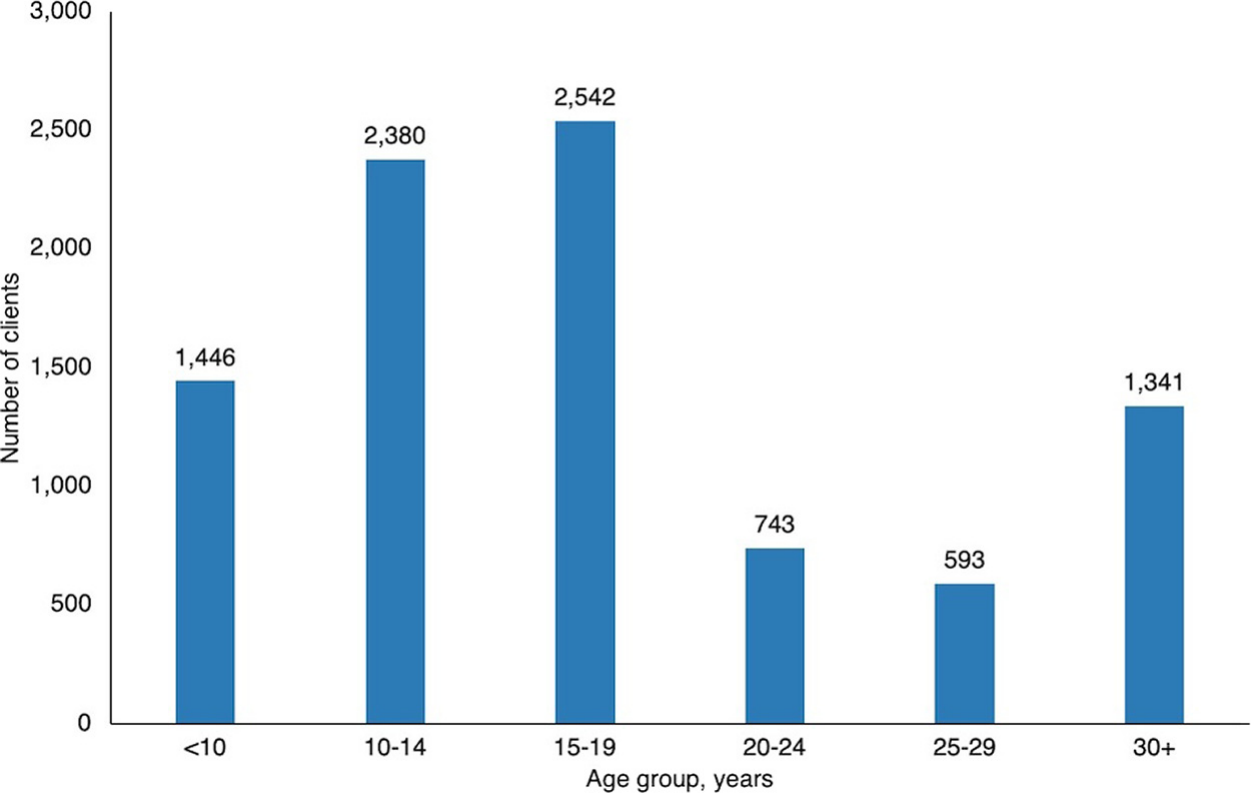
Gender-Based Violence Cases in All Lighthouse Trust Clinics, by Age Group, January 2020–June 2024, Malawi

From January 2020 to June 2024, 5 Lighthouse Trust COEs (Lighthouse Center, Martin Preuss Center, Umodzi Family Center, Rainbow Clinic, and Tisungane Clinic) and 2 supported health centers (Kawale and KCH) reported 9,045 cases of GBV among individuals aged younger than 10 years to older than 30 years. The majority of GBV cases were reported among adolescents aged 10–19 years (54%) (Figure 2). Approximately 92% of survivors were female.

**FIGURE 3:**
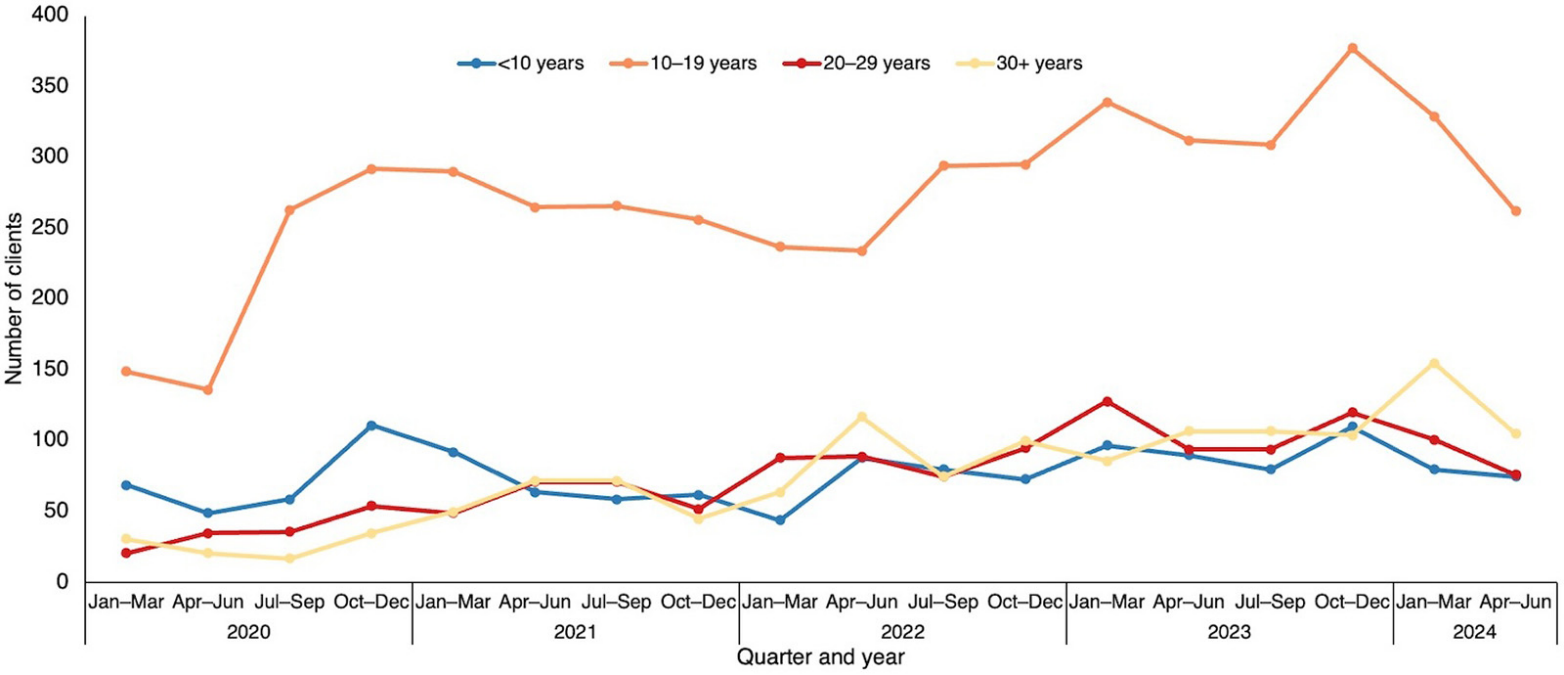
Gender-Based Violence Trends by Gender, Lighthouse Trust Clinics, January 2020–June 2024, Malawi

**FIGURE 4:**
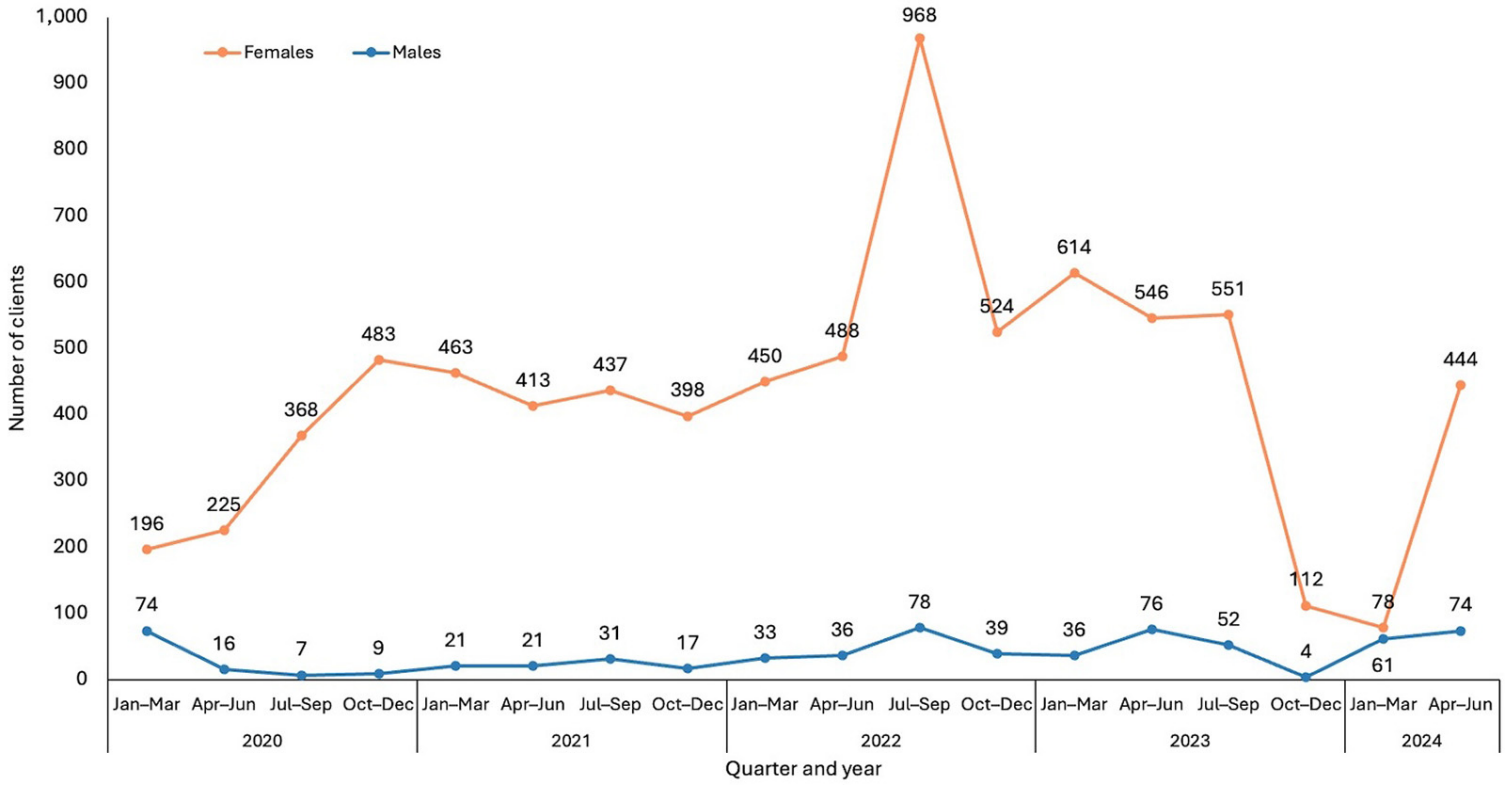
Gender-Based Violence Case Trends by Age Group, Lighthouse Trust Clinics, January 2020–June 2024, Malawi

The number of GBV survivors identified and managed increased from January 2020 to September 2023, especially among adolescent survivors aged 10–19 years (Figure 3). Cases among men remained consistently low (Figure 4). A notable sharp increase among females in reported cases occurred from July to September 2022, coinciding with LIVES training on GBV identification and intimate partner violence reporting for all HIV diagnostic assistants, psychosocial counselors, and nurses. During this period, a session with the police also aimed to enhance community sensitization activities. Conversely, a significant drop in cases between October 2023 and March 2024 was attributed to a decline in provider screening due to the heavy workload burden. Following a review meeting to address these issues, providers were encouraged to resume regular screening, which led to an upward trend in reported cases from April to June 2024.

The number of GBV survivors identified and managed increased from January 2020 to September 2023, especially among adolescent survivors aged 10–19 years.

### Gender-Based Violence by Type

**FIGURE 5:**
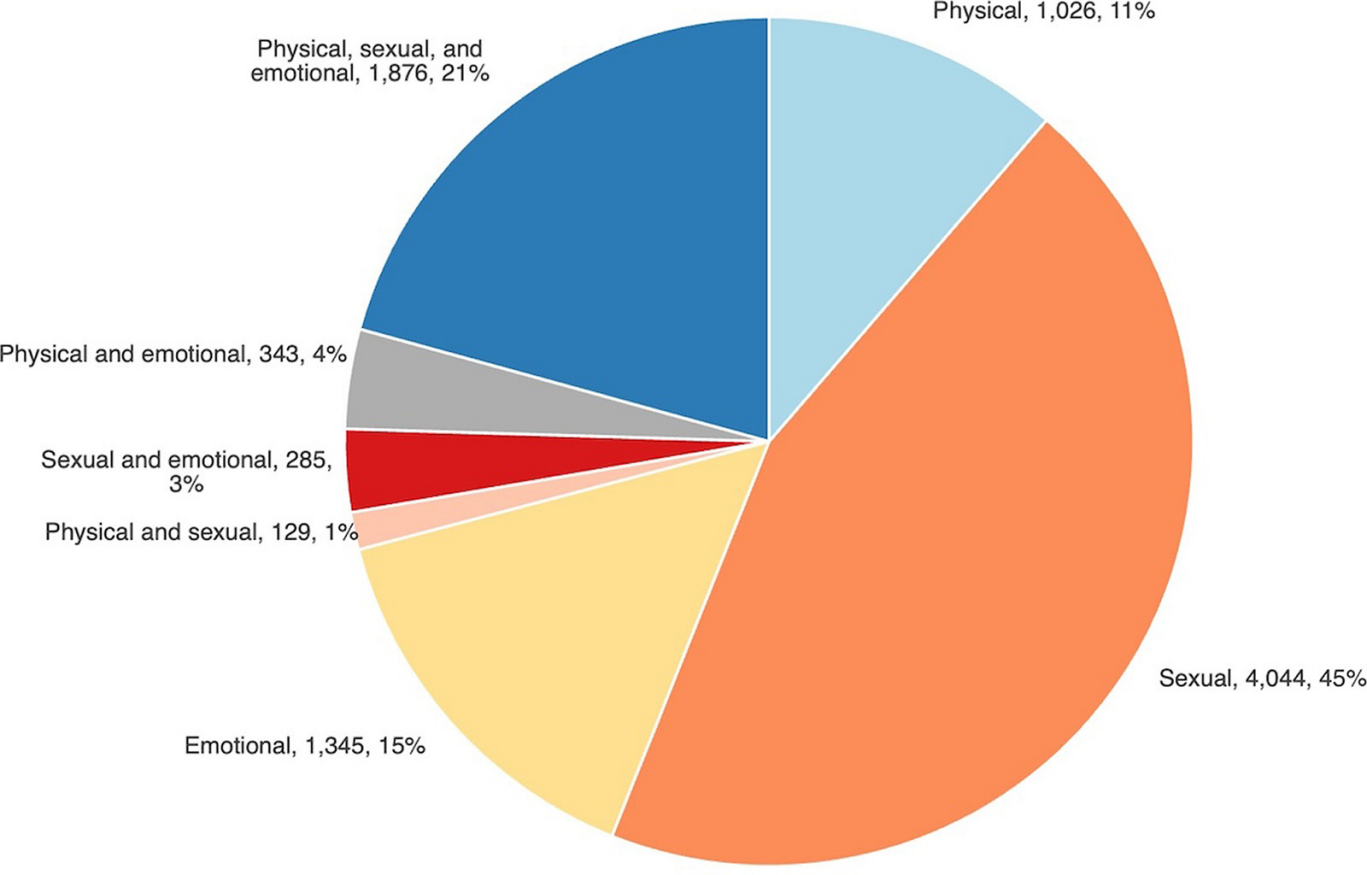
Gender-Based Violence Cases by Violence Type, Lighthouse Trust Clinics, January 2020–June 2024, Malawi

**FIGURE 6:**
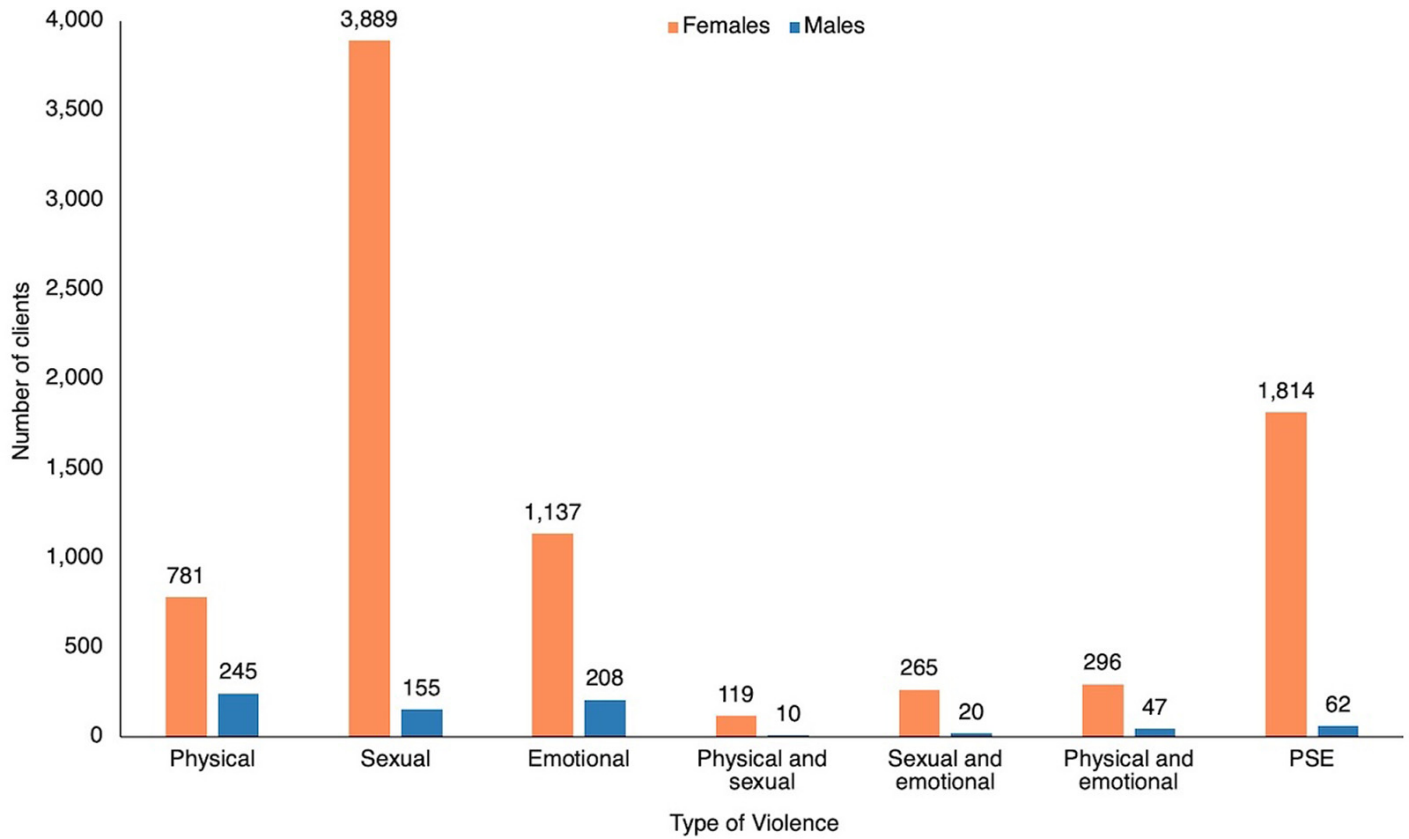
Gender-Based Violence Cases by Gender and Violence Type, Lighthouse Trust Clinics, January 2020–June 2024, Malawi Abbreviation: PSE, physical, sexual, and emotional.

The type of violence reported included physical, sexual, physical, and emotional. In addition, a combination of violence types were also reported, such as physical and sexual; sexual and emotional; physical and emotional; and physical, sexual, and emotional (PSE). The most common form of violence reported was sexual violence, with 4,044 cases (45%), and the least common was the combination of physical and sexual violence, with 129 cases (1%) (Figure 5). Physical violence was the most frequently reported type of GBV for males (N=245), followed by emotional (N=208), while sexual violence was the predominant form reported by females (N=3,889) (Figure 6).

### Post-Gender-Based Violence Care Services

**FIGURE 7:**
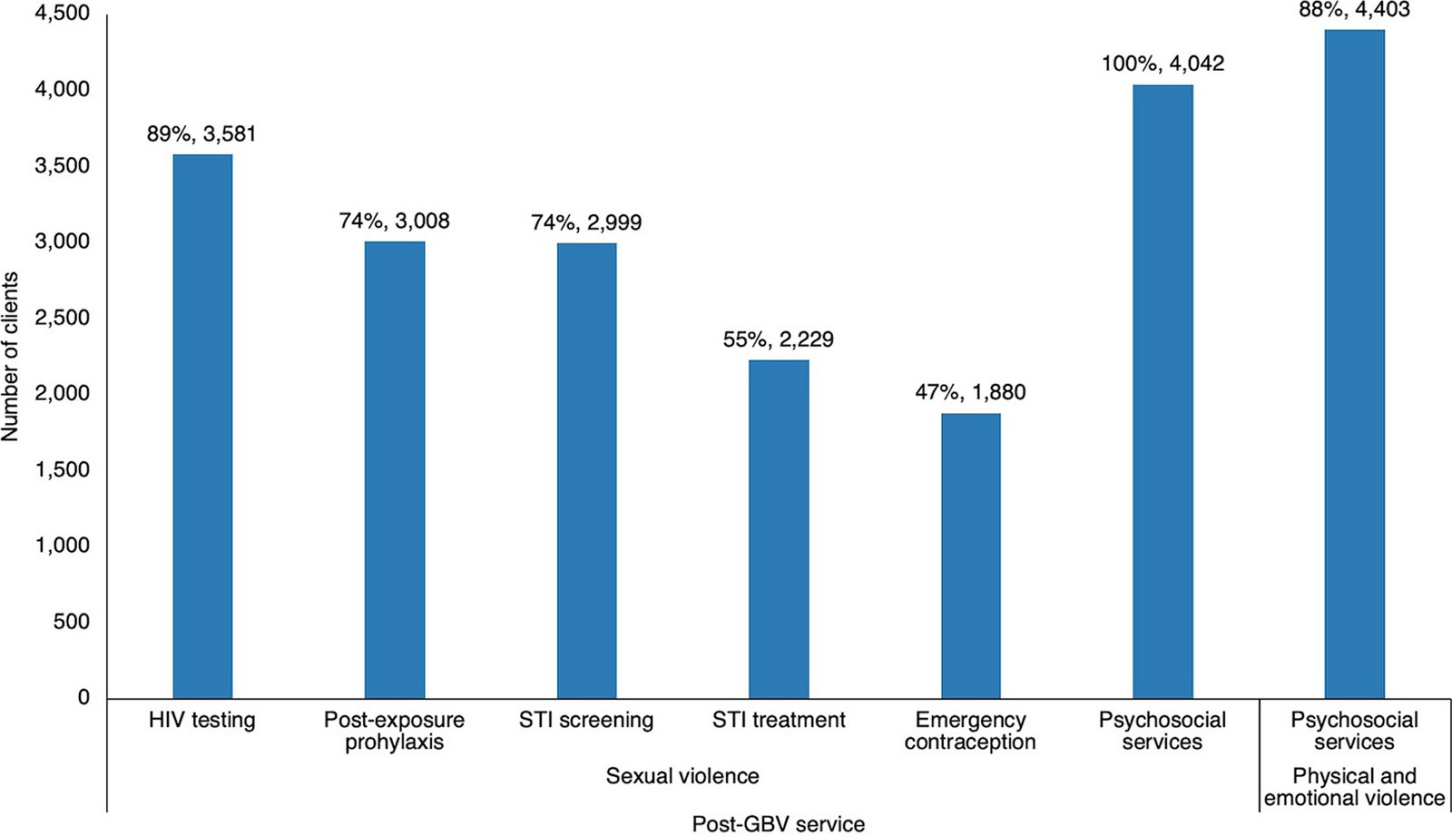
Post-GBV Care Services Provided to Survivors, Lighthouse Trust Clinics, January 2020–June 2024, Malawi Abbreviations: GBV, gender-based violence; STI, sexually transmitted infection.

A total of 18,100 post-GBV services were documented. One survivor may receive more than 1 type of service depending on their needs. Among all survivors who experienced sexual violence, the most common type of service that was provided was PSS, where all (100%) of clients received this service, followed by HIV testing (89%), STI screening (74%), and PEP (24%). Among those who experienced physical and emotional violence, 88% received PSS (Figure 7).

### Violence Perpetrators

There were 3,804 cases where the perpetrators of violence were reported as either known (parent, intimate partner, or other known individual such as neighbor or relative) or unknown to the survivor. The majority (44%) of the perpetrators of violence were other known assailants to the GBV survivor, followed by an intimate partner (34%), unknown assailant (12%), and parents (10%) (Table).

**TABLE.** Assailant Identity by Type of Violence, Lighthouse Trust Clinics, January 2020–June 2024, Malawi

	**Type of Violence, No. (%)**							**Total, No. (%)**
	**Physical**	**Sexual**	**Emotional**	**Physical and Sexual**	**Sexual and Emotional**	**Physical and Emotional**	**PSE**	
Parents	50 (7)	70 (5)	185 (30)	3 (2)	15 (15)	21 (12)	27 (5)	372 (10)
Intimate partner	392 (53)	368 (25)	280 (46)	75 (56)	40 (39)	114 (63)	32 (6)	1,304 (34)
Other known	256 (35)	787 (53)	133 (22)	43 (32)	44 (43)	39 (22)	385 (69)	1,689 (44)
Unknown	40 (5)	252 (17)	12 (2)	13 (10)	3 (3)	7 (4)	112 (20)	439 (12)
Total, no.	738	1,477	610	134	102	181	556	3,804

Abbreviation: PSE, physical, sexual, and emotional.

Intimate partners constituted the largest proportion of assailants in cases of physical violence (53%), emotional violence (46%), combined physical and sexual violence (56%), and combined physical and emotional violence (63%). In contrast, other known assailants were the primary perpetrators of sexual violence (53%), combined sexual and emotional violence (43%), and PSE violence (69%). The highest violence type perpetrated by parents was emotional violence, representing 30% of emotional violence cases. Although the percentage of violence by unknown assailants was relatively low, they still contributed notably to PSE violence (20%) and sexual violence (17%).

## DISCUSSION

We described integrating GBV services into routine HIV clinic operations of Lighthouse Trust, Malawi’s largest HIV services provider. We outlined how Lighthouse Trust effectively integrated GBV care into HIV services by developing and implementing standardized procedures for screening, identification, care, referral, and documentation to optimally support GBV survivors. These services were further strengthened by strong collaboration with the OSCs. We noted significant reported GBV cases affecting adolescents aged 10–19 years. This trend is in line with broader findings, such as those from the Violence Against Children and Young Women Survey,^5^ revealing widespread violence among this demographic, with sexual violence reported most frequently. We documented a marked rise in GBV reports since July 2020, coinciding with the COVID-19 pandemic. Though there are no definite figures, some nongovernmental organizations reported that GBV incidents against women and girls aged 15–49 years increased in 2020, prompting the Malawi President to announce stricter penalties for perpetrators.^31^^,^^32^ The observed increase in GBV reporting aligns with global trends, exacerbated by the COVID-19 pandemic,^33^ indicating the pervasiveness of the issue and the critical need for such integrated care models.^34^

Consistent with global data,^35^ our findings show a disproportionate number of female survivors. However, this difference may not fully capture the prevalence of GBV, as studies suggest that men may be less likely to report such experiences due to factors like social stigma and gender norms.^36^^,^^37^

The integration of GBV services into HIV care has led to a more holistic approach to client care and has the potential to improve client retention and overall outcomes. Clients reported feeling more supported and were more likely to engage in continuous care.

The integration of GBV services into HIV care has led to a more holistic approach to client care and has the potential to improve client retention and overall outcomes.

The majority of GBV survivors at Lighthouse Trust received psychosocial support alongside STI screening and HIV testing. For the clients that received PEP, documented completion rates were low (<20%) because many did not return to the clinic for their follow-up visit or were unreachable via the phone number provided. Seroconversion rates were not documented; however, Lighthouse Trust follows Ministry of Health guidelines for antiretroviral therapy (ART) initiation, where all clients who tested HIV positive were actively linked to ART at the clinic. Notably, most assailants were known to the survivors but were neither parents nor intimate partners, with the latter constituting the second most frequent category of perpetrators. The high rates of known assailants are in concert with what others have reported^38^^–^^40^ and highlight the critical need for secure refuge options for survivors, emphasizing the role of safe houses in GBV response frameworks.^22^

## LESSONS LEARNED

Since 2020, Lighthouse Trust has integrated post-GBV care into its services, standardizing care through standard operating procedures and training health care providers to screen and care for GBV survivors in a manner that would not retraumatize GBV survivors, aligning with findings from similar settings.^18^^,^^22^ The training also expanded providers’ recognition of emotional violence, leading to a more comprehensive approach to addressing all forms of GBV.

The Lighthouse Trust model, characterized by its multidisciplinary team approach and survivor-centered care, proved effective in our setting and could serve as a replicable model for other clinics seeking to integrate GBV services. The key to its success lies in strong stakeholder collaboration and ongoing training and capacity-building. Police involvement in the clinic setting can be beneficial when handled sensitively. In addition, continued community sensitization will ensure increased awareness of GBV and access to post-GBV services.

Challenges in documenting multiple types of GBV, such as combined physical, sexual, and emotional violence, were noted, leading to potential underreporting or misreporting. Additionally, the GBV register’s focus was primarily on younger age groups, inadvertently resulting in the 30+ age group being grouped together without further disaggregation. Implementing an electronic data management system could effectively address documentation challenges by enabling accurate tracking of multiple GBV types, linking GBV care to HIV outcomes, and improving data capture across all age groups.

Furthermore, guardians sometimes hesitated to report sexual violence against child survivors to the police. In some cases, children were given money for sexual encounters with adults, mistakenly leading guardians to view the interactions as consensual, not considering the minors’ inability to consent legally. There were also cases where guardians were reluctant to report a child’s GBV experience if they were financially dependent on the perpetrator. This emphasizes an urgent need for increased community awareness about GBV affecting children.^22^ In addition, the lack of a safe house further complicated situations where child survivors could not be safely housed with relatives, highlighting the need for secure shelters for minors who are survivors of GBV.

Heavy workloads contributed to incomplete documentation, suggesting that the actual number of GBV cases may be higher than reported, as well as inconsistent proactive GBV screening and gaps in necessary support service referrals, with some cases prematurely reported to the police without the survivors first receiving health care intervention. Training staff to manage workloads more efficiently and emphasizing the importance of comprehensive documentation could help ensure no cases are overlooked. However, this would require additional resources, which may be difficult to secure in a resource-limited setting like Malawi.

Despite making strides in integrating GBV into routine care, we cannot attribute the impact of post-GBV services to ART retention, viral load suppression, or other HIV-related outcomes. Pre- and post-integration analysis of the survivors would also be a challenge as documentation tools for GBV and HIV do not have a linking identifier.

Community sensitization efforts and GBV screening at ART clinics have contributed to an increase in reported GBV cases, though some clients may not have received the complete post-GBV treatment package if they reported to the facility beyond the recommended time, particularly those who required PEP and emergency contraceptives.

The COVID-19 pandemic underscored the critical importance of maintaining adaptable and resilient service delivery models, particularly when OSCs were temporarily converted into COVID-19 isolation centers and survivors were compelled to seek help in overcrowded hospitals. This shift may have deterred some from accessing services, highlighting the need for contingency planning in public health crises. Despite these challenges, the continued increase in reported GBV cases during the pandemic emphasizes the persistent need for accessible, survivor-centered care, even under strained circumstances.

## RECOMMENDATIONS

In conclusion, effective GBV care is vital for HIV clients, with provider training in LIVES enhancing the identification and support of GBV survivors. The large number of reported GBV cases in our findings highlights the need for such interventions. Though not explicitly analyzed here, the prevalence of GBV cases likely influences HIV outcomes among people living with HIV and those accessing HIV prevention services. Delivering comprehensive GBV care requires raising awareness among providers and the community and ensuring survivors receive comprehensive GBV care, with efforts made to ensure service accessibility, confidentiality, and safety. Collaboration within health facilities and across governmental ministries—such as health, gender, education, and judicial sectors, is essential for streamlined GBV awareness, prevention, care, and support, including the establishment of safe houses for GBV survivors (especially children) who experienced violence in their homes. Additionally, implementing an electronic medical records system specifically designed to capture GBV-related data linked with HIV reporting systems, comprehensive provider training, and a robust monitoring and evaluation framework will strengthen GBV services in ART clinics, fostering a culture prioritizing all clients’ safety and dignity.

## Notes

**Peer Reviewed**
